# Prolonged culturing of colonic epithelial organoids derived from healthy individuals and ulcerative colitis patients results in the decrease of LINE-1 methylation level

**DOI:** 10.1038/s41598-024-55076-8

**Published:** 2024-02-23

**Authors:** Ruta Inciuraite, Ruta Steponaitiene, Odeta Raudze, Ugne Kulokiene, Vytautas Kiudelis, Rokas Lukosevicius, Rasa Ugenskiene, Kestutis Adamonis, Gediminas Kiudelis, Laimas Virginijus Jonaitis, Juozas Kupcinskas, Jurgita Skieceviciene

**Affiliations:** 1 Institute for Digestive Research, Academy of Medicine, Lithuanian University of Health Sciences, A. Mickeviciaus St. 9, 44307 Kaunas, Lithuania; 2 Department of Gastroenterology, Academy of Medicine, Lithuanian University of Health Sciences, A. Mickeviciaus St. 9, 44307 Kaunas, Lithuania; 3 Department of Genetics and Molecular Medicine, Academy of Medicine, Lithuanian University of Health Sciences, A. Mickeviciaus St. 9, 44307 Kaunas, Lithuania

**Keywords:** DNA methylation, Ulcerative colitis, Gastrointestinal models

## Abstract

Patient-derived human intestinal organoids are becoming an indispensable tool for the research of digestive system in health and disease. However, very little is still known about the long-term culturing effect on global genomic methylation level in colonic epithelial organoids derived from healthy individuals as well as active and quiescent ulcerative colitis (UC) patients. In this study, we aimed to evaluate the epigenetic stability of these organoids by assessing the methylation level of LINE-1 during prolonged culturing. We found that LINE-1 region of both healthy control and UC patient colon tissues as well as corresponding epithelial organoids is highly methylated (exceeding 60%). We also showed that long-term culturing of colonic epithelial organoids generated from stem cells of healthy and diseased (both active and quiescent UC) individuals results in decrease of LINE-1 (up to 8%) methylation level, when compared to tissue of origin and short-term cultures. Moreover, we revealed that LINE-1 methylation level in sub-cultured organoids decreases at different pace depending on the patient diagnosis (healthy control, active or quiescent UC). Therefore, we propose LINE-1 as a potential and convenient biomarker for reliable assessment of global methylation status of patient-derived intestinal epithelial organoids in routine testing of ex vivo cultures.

## Introduction

The term ‘organoid’ refers to cells growing in a defined three-dimensional (3D) environment in vitro that form clusters of cells capable of self-organization and differentiation into functional cell types^1^, and mirroring the structure and functions of an in vivo organ^2^. The mini-gut culture system, referred as human intestinal epithelial organoid^3^, derived from highly Lgr5 (Leucine-rich repeat-containing G-protein coupled receptor 5) expressing intestinal stem cells^4^ is one of the most widely used model systems for a broad range of scientific applications, including, but not limited to modeling of intestinal diseases pathogenesis mechanisms (such as ulcerative colitis (UC)), tissue development research, development of new treatment tools for personalized medicine, etc.^5–7^.

Epigenetic processes, such as global DNA methylation, gene-specific DNA methylation, modifications of histone proteins and chromatin, etc., are crucial in regulating gene expression, development, maintaining and transforming genome stability and genomic integrity in health and disease (incl. cancer and inflammatory diseases)^8,9^. Long Interspersed Nucleotide Element 1 (LINE-1) is a widely accepted universal surrogate genomic DNA methylation marker correlating with the global DNA methylation levels^10^. In normal state LINE-1 CpG content is hypermethylated^11^ and under specific circumstances, such as disease development (various cancers, autoimmune diseases, etc.), harsh external influences, the level of methylation within LINE-1 elements decreases^12–14^. Accordingly, previously conducted study focusing on DNA methylation in UC reveals the relation between colon tissue inflammation and hypomethylated DNA spectrum^15^.

UC, a state referred as idiopathic chronic, progressive immune-mediated inflammatory bowel disease (IBD) characterized by fluctuating periods of mucosal inflammation activity followed by phases of endoscopic remission and mucosal healing^16,17^ was extensively studied in the context of epigenetic and genetic background. Panels of differentially expressed genes in correlation with methylation patterns related to UC were identified (summarized by Annese^18^ and Gould et al*.*^19^), however alterations of genome-wide methylation in repetitive transposable elements in UC is still scarcely explored.

The importance of methylation alterations in ageing and regional identity of intestinal epithelial organoid system has been studied on regional and genome-wide levels. A very recent study by Edgar et al*.* also showed that genome-wide methylation status of intestinal epithelial organoids cultured for an extended period of time decreases^20^. However, up to date most of the epigenetic studies were conducted using murine intestinal organoid models, or healthy human intestinal organoid models, whereas studies analyzing the epigenetic stability of epithelial organoids derived from UC patients are still lacking.

The aim of current study was to evaluate the epigenetic stability of human intestinal epithelial organoids obtained and cultured from colonic biopsies of UC patients and healthy controls during long-term culture using quantitative methylation level of LINE-1 as a surrogate global genomic methylation marker. Our study design allowed us to not only evaluate whether the generated ex vivo epithelial organoid model systems have a stable DNA methylation profile compared to their original source (colon biopsy and colonic crypts) in the context of both active and quiescent UC, and healthy control conditions, but also let us to assess if long-term culturing of these healthy- and diseased patients-derived epithelial organoids introduces the changes in LINE-1 methylation intensity. Collectively, our study allowed us to fill the knowledge gap regarding dynamics of LINE-1 methylation in the UC patient-derived colon epithelial organoids cultured for prolonged time.

## Results

### Healthy- and UC patient-derived colonic epithelial organoids exhibit stable characteristic morphological phenotype during long-term culturing

3D epithelial organoids of control (CON) individuals as well active (aUC) and quiescent (qUC) ulcerative colitis patients were grown in primary culture for 7–14 days before passaging. The growth dynamic of patient-derived colonic organoids was assessed microscopically, observing the transition from freshly isolated crypts (day 0 in culture) to small (5–8 days in culture) and large (8–14 days in culture) cystic structures in all study groups. Neither the patient diagnosis, nor the duration of cultivation affected the cell behavior, or the microscopic appearance of intestinal epithelial organoids and typical cystic appearance of colonic organoids was retained in high-passage number (Passage 5) cultures (Supplementary Fig. 1A). Additionally, immunofluorescence characterization of generated organoids confirmed the proper polarity of organoid-forming epithelial cells, assessed as the basolateral expression of β-catenin and apical expression of F-actin (Phalloidin) (Supplementary Fig. 1B), defining the central lumen. Furthermore, undifferentiated patient-derived 3D epithelial organoids also mimicked the cellular composition and architecture of human colon epithelium. High expression of proliferating cell marker Ki-67, as well as tight junction protein ZO-1 was observed, while the levels of specialized intestinal cell markers (colonocytes—Cytokeratin 20, Goblet cells—Mucin 2, and enteroendocrine cells—Chromogranin A) were comparably lower (Supplementary Fig. 1C–G).

Our results, namely the timing of epithelial organoid formation, epithelial polarization, and cellular composition, are in agreement with studies by other research groups, where they also described adult human stem cell-generated ex vivo experimental models for studies of the intestinal system^21,22^.

### Healthy and diseased human colon tissues and respective epithelial organoids have a highly methylated LINE-1 region

**Figure 1:**
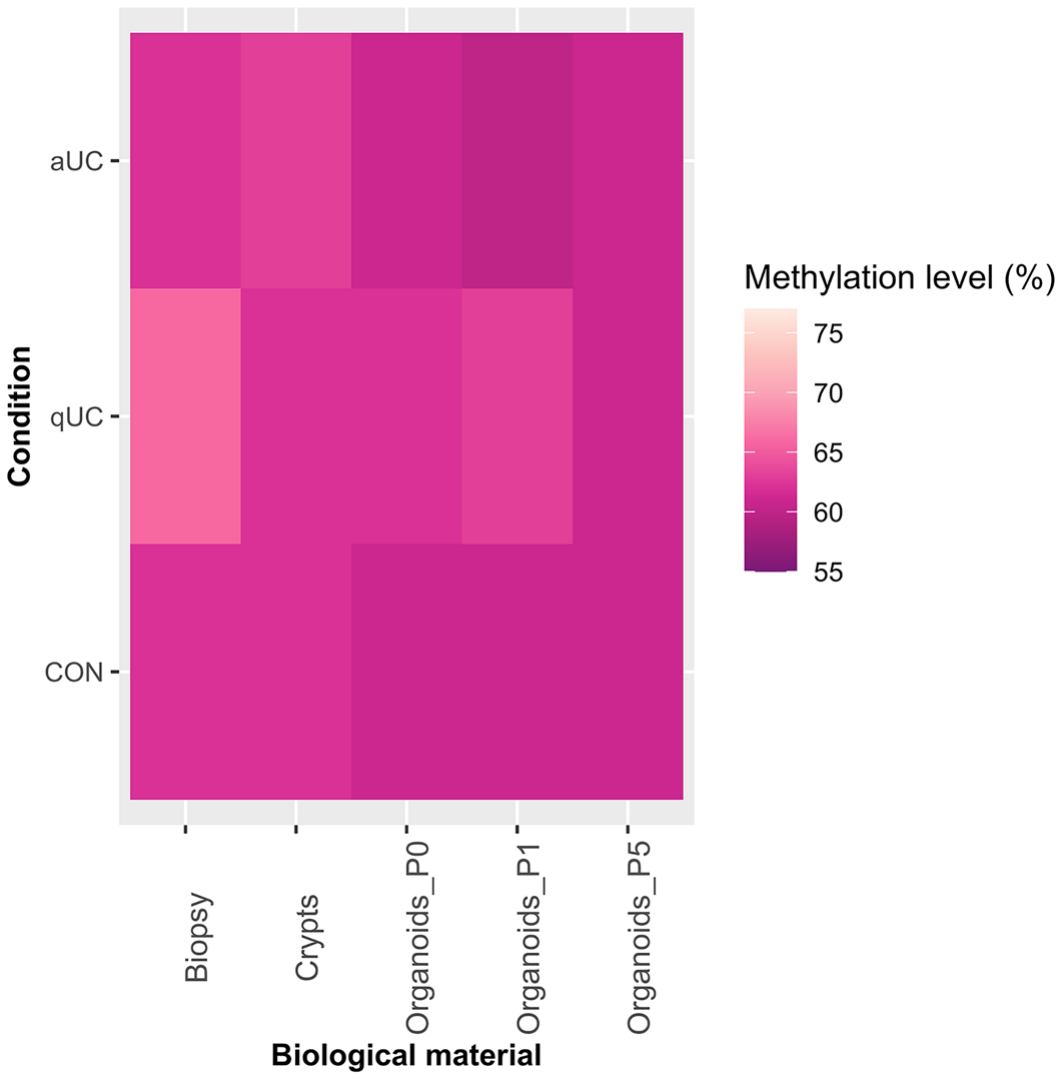
Heatmap showing average LINE-1 region methylation level of different biological samples in UC patients with active and quiescent disease and control subjects. The colour of the box represents the average methylation level (%) in each study group. CON—control (n = 6), qUC (n = 7)—quiescent ulcerative colitis, aUC (n = 6)—active ulcerative colitis.

Quantitative evaluation of LINE-1 region methylation level was performed in the control (CON), active (aUC) and quiescent (qUC) ulcerative colitis groups at each point of the biological sample studied—colon biopsies, isolated crypts, primary epithelial organoids (P0), short-term (P1) and long-term (P5) cultured epithelial organoids. The presented methylation estimates show that the LINE-1 region was highly methylated in all studied biological sample groups for all conditions (Fig. 1). The methylation level of our selected LINE-1 region varied at certain level both when comparing different study groups (CON, aUC, qUC) and between different biological samples (biopsies, crypts, organoids) (Table 1). The observed average (± SD) percentage values of LINE-1 methylation level in the entire study cohort ranged between 69.4 ± 2.9% (in the group of qUC Biopsy) and 61.8 ± 3.8% (in the group of aUC Organoids_P1).

**Table 1:** The summary table of average LINE-1 region methylation level in study cohort.

	CON	Active UC	Quiescent UC
Biopsy, %			
Mean ± SD	68.7 ± 4.3	66.7 ± 4.4	69.4 ± 2.9
Crypts, %			
Mean ± SD	68.6 ± 4.9	66.3 ± 4.6	67.9 ± 3.7
Organoids_P0			
Mean ± SD	66.9 ± 4.1	65.6 ± 4.2	66.0 ± 3.9
Organoids_P1			
Mean ± SD	66.5 ± 5.4	61.8 ± 3.8	65.5 ± 3.7
Organoids_P5			
Mean ± SD	60.6 ± 2.3	62.6 ± 4.8	64.4 ± 3.9

SD—standard deviation, CON—control, UC—ulcerative colitis.

Average (± SD) LINE-1 methylation level was almost the same in the colon biopsy samples of control and qUC groups and reached 68.7 ± 4.3% and 69.4 ± 2.9% (*p*_adj._ = 0.61), respectively, while it was slightly lower in the group of aUC (66.7 ± 4.4%, *p*_adj._ = 0.47 and *p*_adj._ = 0.15, compared to CON and qUC, respectively). The observed percentage methylation values in the colonic crypts showed the same tendency as in the biopsies, being more similar between CON and qUC groups (68.6 ± 4.9% and 67.9 ± 3.7%, *p*_adj._ = 0.59) and lower in the aUC group (66.3 ± 4.6, *p*_adj._ = 0.52 and *p*_adj._ = 0.52, compared to CON and qUC, respectively). Finally, primary cultures of generated organoids also followed the trend and revealed minor decrease in LINE-1 methylation in all groups, when compared to biopsy samples. The LINE-1 region methylation percentage in CON and qUC groups was 66.9 ± 4.1% and 66.0 ± 3.9% (*p*_adj._ = 1.00), and in aUC group in was 65.6 ± 4.2% (*p*_adj._ = 0.84 and *p*_adj._ = 1.00, compared to CON and qUC, respectively).

Hence, our initial observations did not reach statistical significance but clearly showed similar trends to previous studies that associate active UC with DNA hypomethylation^15^.

### LINE-1 methylation level of healthy and diseased individual-derived colonic epithelial organoids decreases over long-term culturing

**Figure 2:**
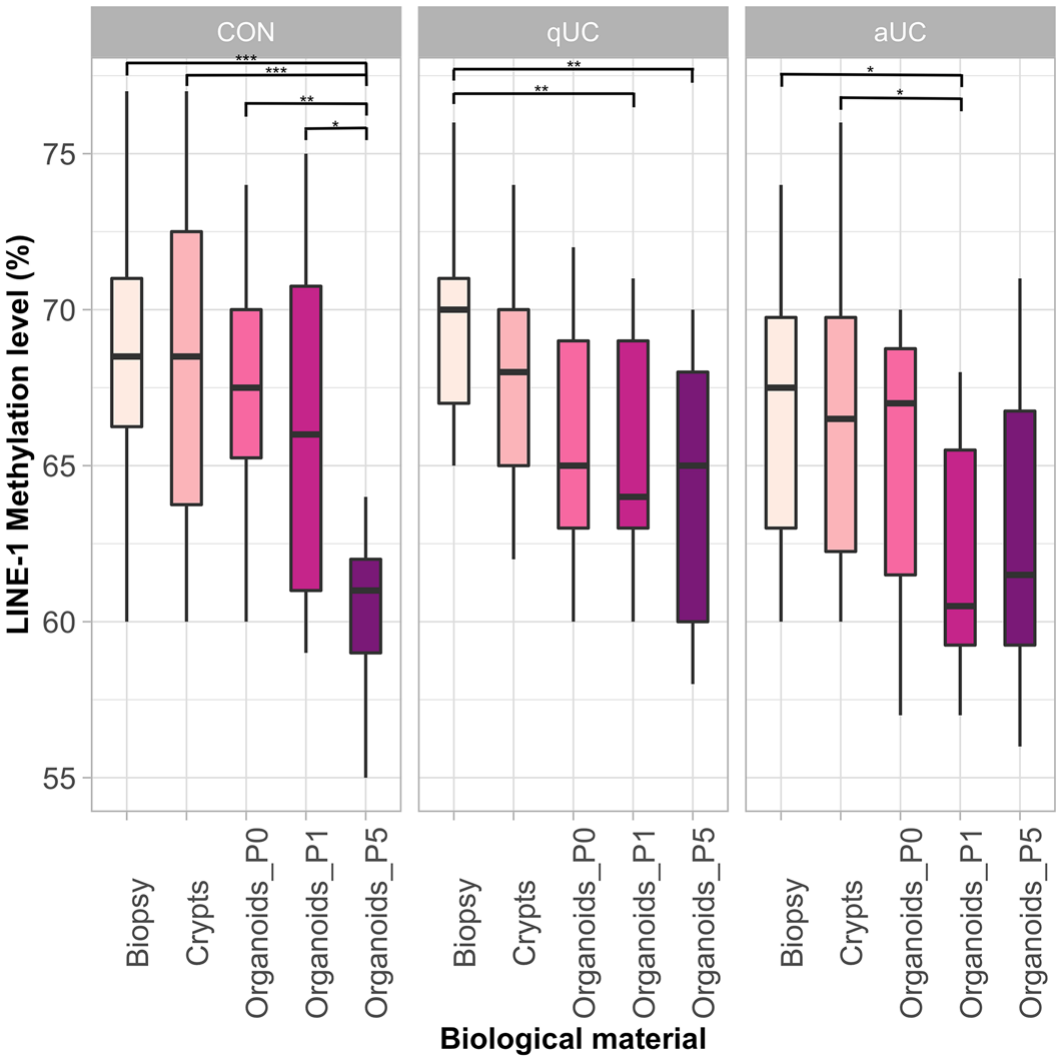
Boxplots showing comparisons of LINE-1 methylation level of different biological samples in healthy controls and patients with active and quiescent UC. The colour of the boxes represents different biological material. Distinct panels correspond to different health conditions. CON—control (n = 6), qUC (n = 7)—quiescent ulcerative colitis, aUC (n = 6)—active ulcerative colitis. *p_adj._ ≤ 0.05, **p_adj._ ≤ 0.01, ***p_adj._ ≤ 0.001.

Analysis of DNA methylation level revealed that methylation level of the initial colon biopsy samples in all study groups (control/active/quiescent UC) differed significantly compared to the respective pure epithelial colon organoid cultures (Fig. 2). In the control group (CON) representing the healthy colon, LINE-1 methylation level of late-passage organoids (P5) decreased significantly compared to colon biopsy (8.1%, *p*_adj._ = 1.04 × 10^–4^), crypts (8.0%, *p*_adj._ = 2.48 × 10^–4^), and early-passage P0 (6.3%, *p*_adj._ = 2.00 × 10^–3^) and P1 organoids (5.9%, *p*_adj._ = 0.019). Significant differences, in terms of LINE-1 methylation level, were also observed when comparing P1 and P5 epithelial organoids generated from patients with quiescent UC (qUC) to primary tissue, i.e., biopsies. In this group, methylation level of analyzed LINE-1 region dropped down by approx. 4.0% (*p*_adj._ = 6.00 × 10^–3^) and 5.0% (*p*_adj._ = 1.00 × 10^–3^) in P1 and P5 organoids, respectively, when compared to biopsy samples. Similar observations were made in group of patients with active UC (aUC), where LINE-1 methylation level of P1 organoids decreased significantly by approx. 4.9% and 4.6% when compared to colon biopsy (*p*_adj._ = 0.012) and crypts (*p*_adj._ = 0.019) samples, respectively. Analogous trend of considerable LINE-1 methylation level decrease (by approx. 4.0%) was also noticed in group of late-passage epithelial organoids (P5) of aUC patients compared to respective colon tissue samples, however, differences did not reach the statistical significance (*p* = 0.102).

Altogether, our results reveal that establishment and long-term culturing of colonic epithelial organoid cultures derived from both healthy controls and UC patients is associated with epigenetic changes, namely the LINE-1 hypomethylation.

### The LINE-1 methylation dynamic differs in epithelial organoids generated from healthy control, and healed and inflamed colon of UC patients

**Figure 3:**
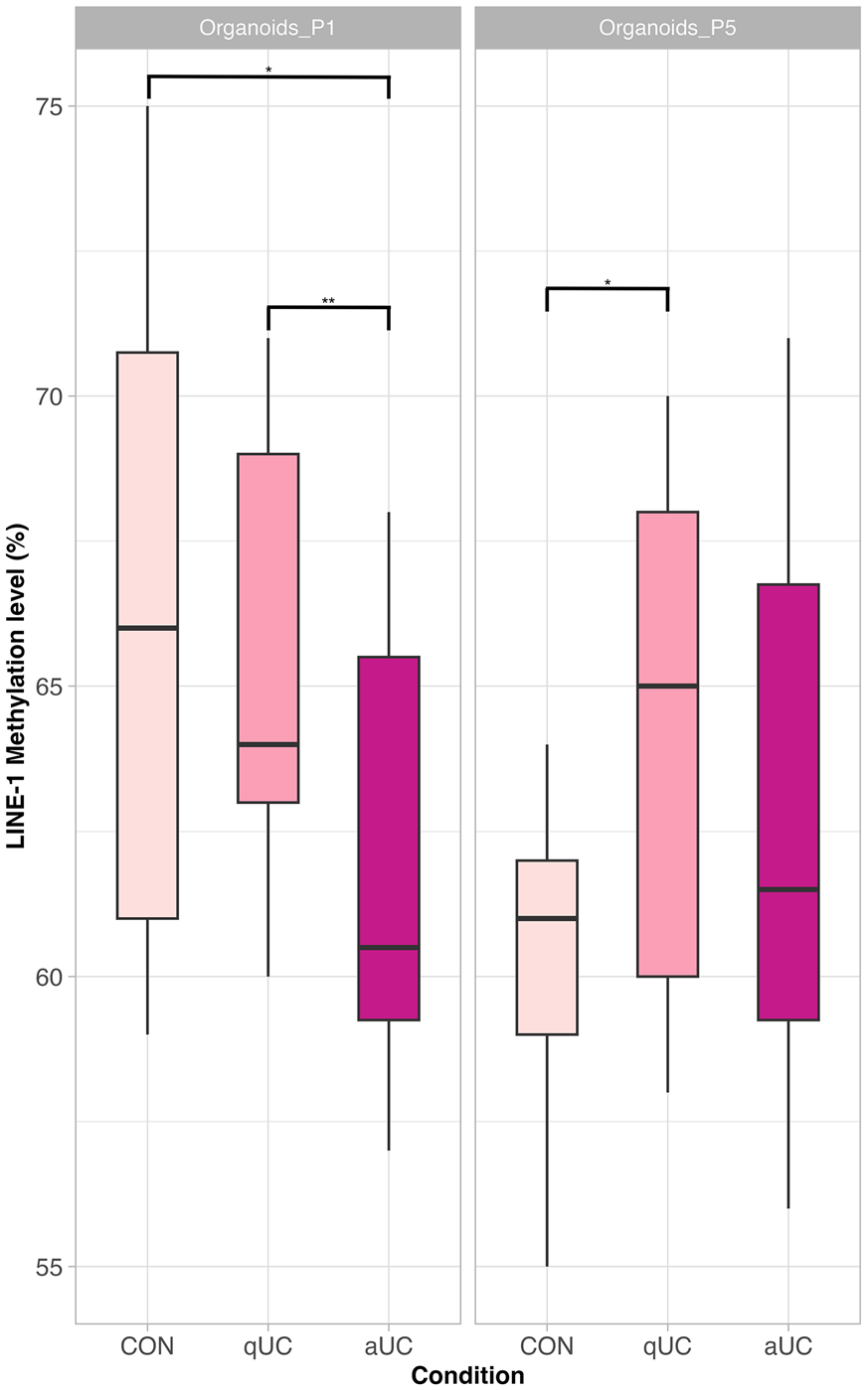
Boxplots comparing LINE-1 methylation level in early- and late-passage epithelial organoids between healthy controls and patients with active and quiescent UC. The colour of the boxes represents different health conditions. Distinct panels correspond to different organoid passage number. CON—control (n = 6), qUC (n = 7)—quiescent ulcerative colitis, aUC (n = 6)—active ulcerative colitis. *p_adj._ ≤ 0.05, **p_adj._ ≤ 0.01.

Additionally, our study design also allowed us to evaluate whether the changes in DNA methylation intensity differ in sub-cultured epithelial organoids depending on the colon inflammation activity. LINE-1 methylation level comparisons of early- and late-passage organoids-derived data revealed significant differences between health conditions (Fig. 3). Methylation level of LINE-1 region was lower by approx. 4.7% and 3.7% in the P1 organoids of aUC patients when compared to either CON (*p*_adj._ = 0.018) or qUC (*p*_adj._ = 0.010) group, respectively. Interestingly, sizable decrease in LINE-1 methylation level (reaching the value of aUC group) was observed in the late-passage (P5) organoids of CON group, which, in turn, resulted in significant difference (by 3.8%) when compared to qUC P5 organoids (*p*_adj._ = 0.024).

To sum up, our results suggest that long-term culturing of colonic epithelial organoids of healthy individuals and active and quiescent UC patients results in different pace of LINE-1 methylation decrease.

## Discussion

Since the development of human intestinal epithelial cell-derived organoids over a decade ago^23^, their use as powerful translational research tool to study intestinal epithelial cell biology and pathophysiology has continued to expand rapidly. However, in vitro culturing of intestinal organoids lacks in vivo environment, such as gut microbiota or signals from other cell types. Therefore, some of their initial characteristics may be altered due to prolonged culturing which is required not only for expansion and maintaining organoids in culture, but also for specific experiments (e.g., focusing on development, long-term effect of stimulation, etc.). Even though it has already been shown that intestinal organoids maintain genetic stability^24^, very little is known about the epigenetic regulation, especially in the context of IBD. Thus, here we provide the evidence for global DNA methylation level changes in human adult stem cell-derived 3D colonic epithelial organoids during long-term culturing. We not only describe LINE-1 methylation level fluctuations in epithelial organoids representing healthy colon, but also determine changes in the cultures originated from inflamed and non-inflamed colon sites of patients with UC over time.

First, our study provides a detailed characterization of quantitative LINE-1 methylation status across different levels of studied samples—ranging from whole colon biopsy, narrowing the analysis to epithelial cells in colonic crypts and ending with crypt-generated pure colonic epithelial organoids during prolonged (up to 5th passage) in vitro cultivation. We found that LINE-1 region is highly methylated (exceeding 60% in all cases) in human colon tissue, isolated crypts, and epithelial cell-derived early- and late-passage colonic organoids derived from either healthy control patients or patients with active and quiescent UC. High level of LINE-1 methylation in colon tissue also has been revealed in previous reports^14,25,26^. In our study, when assessing all biological material collectively, we determined the highest methylation of LINE-1 in the control group, while hypomethylation was observed in case of active UC. This observation of altered LINE-1 methylation in UC when compared to healthy colon confirms findings of previous studies reporting gene hypomethylation as a signature feature of UC^15^. However, there is some controversy in the literature when comparing global methylation level data (assessed by quantitative LINE-1) to data originating from gene-level methylation profiling studies. For example, in contrast to the reported gene-level specific methylation level decrease during UC^15^, recently Szigeti et al. reported equally high methylation level of LINE-1 region for colon tissue from healthy subjects and IBD patients^14^. What is more, Quintanilla et al. found different LINE-1 methylation levels in normal mucosa across distinct segments of large intestine^26^. Together, these data suggest different but still high genome-wide methylation levels between healthy and inflamed large intestine and its sites. Similarly, results of our present study also demonstrate high LINE-1 methylation level in both healthy and diseased colon tissue and besides that, such readout is in line with the results from previous study of our group, where we found an average 67.17 ± 4.84% methylated LINE-1 region in endoscopically normal colon mucosa^25^.

Most importantly, our study design enabled the determination of the changes in overall DNA methylation level during prolonged culturing of colonic epithelial organoids derived from either healthy control, healed, or inflamed UC patients’ mucosa. We used LINE-1 as a global genome methylation marker, as LINE-1 elements comprise at least 17–18% of the human genome, are generally highly methylated in somatic tissue and their methylation level correlates significantly with genome-wide 5-methylcytosine content^10^. To the best of our knowledge, there are no previous studies where LINE-1 was used as a surrogate index mirroring global DNA methylation status in human intestinal organoid models. We compared not only colon tissue and primary epithelial organoids (P0), but also involved further cultures, i.e., early passage (P1) and late passage (P5) colonic epithelial organoids, which were cultured for approx. 2–3 weeks and 2 months, respectively. This led us to observation that LINE-1 methylation level of the colon crypt-derived epithelial organoids was lower than initial colon biopsy samples used for ex vivo culture establishment. What is more, our data revealed the different patterns in the decrease pace of LINE-1 methylation level, when comparing healthy control subjects and UC patients. In our data LINE-1 methylation level of healthy control colon-derived epithelial organoids tends to decrease more drastically than in active and quiescent UC patients’ groups. Therefore, we suggest that experimental data derived from late-passage organoids of individuals with different diagnosis should be compared with caution. Even though the epigenetic changes in global DNA methylation context in human intestinal epithelial organoid model (healthy or IBD) during short- or long-term *in vitro* culture have already been revealed in several publications^20,27,28^, the reported findings remain ambiguous. For example, in a study which used a small subset of pediatric IBD patients and healthy subjects, authors established ex vivo intestinal epithelial organoids and profiled epigenotype by assessing disease-associated differentially methylated positions (DMPs) and compared them with DNA methylation profile of highly purified intestinal epithelial cells. Distinct disease-specific epigenetic profile was identified in intestinal epithelium of children with IBD, and organoid cultures partially reflected the methylation profile of purified intestinal epithelium^28^. However, the long-term culturing impact on the methylation profile changes was not evaluated within the frames of this study. Another study using microarray technology (Infinium HumanMethylation450 BeadChip), showed that intestinal epithelial organoid cultures generated from biopsies of different intestinal segments maintain gut site-specific genome-wide DNA methylation profile (i.e., site-specific DMPs) during long-term culturing (up to 3 months)^27^. On the other hand, in the most recent large-scale study, including healthy pediatric and adult subjects, authors evaluated global changes in DNA methylation, gene expression and cellular function induced by human intestinal epithelial organoids culturing over time. The results of this study suggested a shifted epigenetic profile in organoids cultured for an extended period^20^. Accordingly, our results fall in concordance with findings of this study and also indicate that LINE-1 methylation level tends to decrease over prolonged cultivation. Together, our study revealed that methylation pattern differs significantly between primary biopsy sample and colonic epithelial organoids culture (i.e., the longer organoids are cultured, the lower LINE-1 methylation level is) regardless the inflamed (aUC) or non-inflamed (qUC, CON) origin of the biopsy, but the pace of the DNA methylation reduction is diagnosis dependent.

To conclude, our results show that LINE-1 region of both healthy control and UC patients colon tissues and corresponding intestinal epithelial organoids is highly methylated and long-term culturing of these organoids results in decrease of LINE-1 methylation level which proceeds at different pace depending on the inflammation status of primary tissue. Therefore, we suggest that LINE-1 region could potentially be used as an additional routine biomarker in epithelial organoid characterization to assess methylation status, as it resembles the global methylation status results published in previous studies.

## Methods

### Patient cohort description and pathology classification

**Figure 4:**
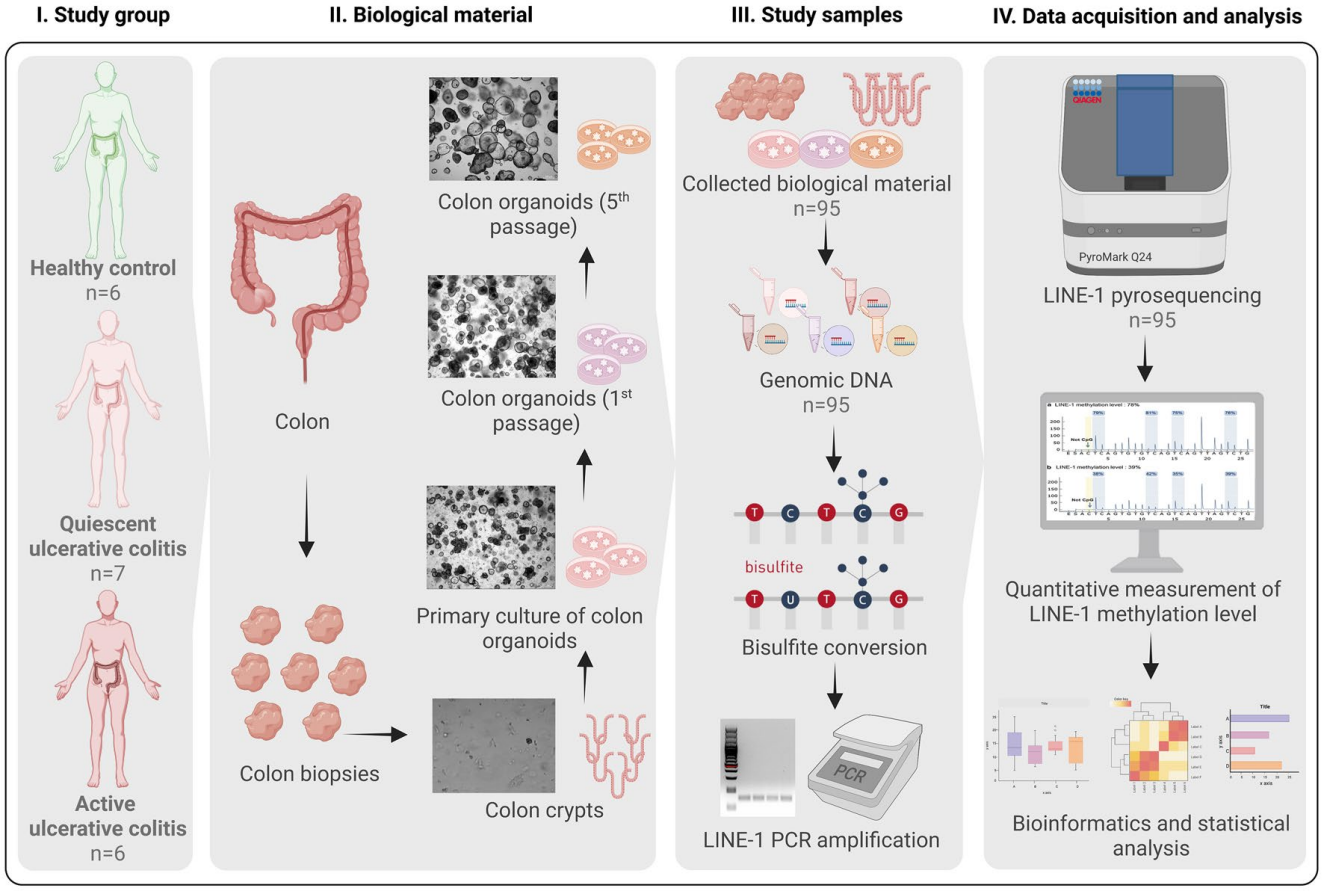
Scheme representing experimental study design and workflow (created with BioRender.com).

The present study was approved by the Kaunas Regional Biomedical Research Ethics Committee (Protocol No. BE-2-31) and written informed consent was obtained from each subject who participated in the study. All research was performed in accordance with relevant guidelines and regulations. This study consisted of three age- and sex-matched groups (Fig. 4). It enrolled 13 age- and sex-matched patients (7 males, 6 females, mean age of patients group 41.9 ± 15.4 years) with a previously established diagnosis of UC based on clinical, endoscopic, and histological examinations, that were scheduled for a colonoscopy either because of a disease flare or for screening purposes. 6 subjects who underwent colonoscopy procedure through colorectal cancer screening program (3 males, 3 females, mean age of control group 53.2 ± 8.5 years) without inflammatory, oncological, or other gastrointestinal diseases were enrolled as controls. Colon biopsy samples from sigmoid colon, rectum*,* or descending colon were obtained during standard colonoscopy procedure from control group individuals and subjects with active UC (aUC) or UC in remission (quiescent—qUC) who were examined at the Department of Gastroenterology, Hospital of Lithuanian University of Health Sciences. Remission of ulcerative colitis was confirmed in patients with stool frequency ≤ 3/day, no rectal bleeding, and healed mucosa at endoscopy (Endoscopic Mayo score ≤ 1). The biopsies for tissue level methylation analysis were immediately flash frozen, whereas biopsies for organoid establishment were placed in DMEM/F-12 medium and processed immediately. Table 2 represents other summarized clinical and demographic data of the study subjects.

**Table 2:** Demographic and clinical characteristics of the subjects.

	CON, n = 6	Active UC, n = 6	Quiescent UC, n = 7
Age			
Mean ± SD	53.2 ± 8.5	43.7 ± 19.3	40.4 ± 12.7
Sex, n (%)			
Female	3 (50.0)	3 (50.0)	3 (43.0)
Full Mayo score			
Min–max	-	4–8	0–2
Biopsy site, n (%)			
Sigmoid colon	3 (50.0)	2 (33.0)	5 (71.00)
Rectum	2 (33.0)	2 (33.0)	1 (14.0)
Descending colon	1 (17.0)	2 (33.0)	1 (14.0)
Extent of UC, n (%)			
Proctitis (E1)	-	0 (0.0)	1 (14.0)
Left-sided colitis (E2)	-	1 (17.0)	2 (28.0)
Extensive colitis (E3)	-	5 (83.0)	4 (57.0)
Previous treatment (Yes/No)			
Immunosuppressants	-	No	Yes
Anti-inflammatory agents	-	Yes	Yes

SD—standard deviation, CON—control, UC—ulcerative colitis.

### Establishment and expansion of human colonic epithelial organoid cultures

3D undifferentiated colonic epithelial organoids from adult intestinal stem cells were established and cultured according to the protocol of IntestiCult Organoid Growth Medium (Human) (OGMH) (06010, StemCell Technologies) with slight adjustments. Briefly, colon biopsies were first minced with sterile scalpel and incubated in Gentle Cell Dissociation reagent (100–0485, StemCell Technologies) to digest colon tissue. After centrifugation and removal of the supernatant, crypts containing intestinal stem cells were removed from biopsies by vigorous pipetting in cold DMEM/F-12 (supplemented with 1% BSA and 15 mM HEPES) medium, passed through a 70 μm pore filter, and the number of isolated crypts was estimated. After centrifugation and removal of the supernatant, isolated colonic crypts were mixed with basement membrane matrix Matrigel (356231, Corning) and seeded into a 24-well cell culture plate, forming 50 μl volume domes. Colon epithelial organoids were cultured in OGMH medium containing factors necessary for structure formation and stem cell renewal, supplemented with antibiotics (penicillin/streptomycin (100 μg/ml) (15140122, Gibco) and the RHO/ROCK signaling pathway inhibitor Y-27632 (for the first two days after seeding). OGMH was changed every 2 days. Colonic organoids were incubated at 37 °C with 5% CO_2_. The growth of undifferentiated 3D colonic organoids was evaluated microscopically (inverted fluorescent microscope ZEISS Axio Observer 7, ZEISS ZEN 3.1 (blue edition) software). The first splitting of the organoid culture was performed after 7–14 days. Each subsequent passage of organoids was performed once the organoids were mature (7–10 days post-passage) until the fifth passage. A portion of fully formed undifferentiated colonic organoids after passage 0, 1, and 5 and portion of initial samples of isolated colon crypts were cryopreserved using CryoStor® CS10 (07930, StemCell Technologies) cell storage reagent. The suspension was transferred to a cryotube and immediately placed at − 80 °C. See Fig. 4 for the overview of study design.

### Microscopical characterization of colonic epithelial organoids

The morphology, cellular composition and functional parameters of the formed 3D intestinal epithelial organoids were evaluated by brightfield and immunofluorescence microscopy. Organoid growth dynamics were monitored daily. For immunofluorescence microscopy, undifferentiated organoids were fixed in 4% paraformaldehyde (1.00496.0700, Sigma-Aldrich) solution, incubated for 30 min, thus releasing them from the *Matrigel matrix*. Further organoid cells were permeabilized with 0.5% Triton-X (9002–93-1, Sigma-Aldrich) solution and blocked with 2% BSA blocking solution. Finally, fluorochrome-conjugated monoclonal antibodies diluted in antibody dilution solution (1:50–1:500) were added to the prepared organoids and incubated for 60 min at RT. Antibodies were applied that are specifically directed against: 1. cell polarity markers (*Anti-beta-catenin-Alexa Fluor 488* (53-2567-42, eBioscience), *F-actin phalloidin-Alexa Fluor 660* (A22285, Invitrogen)); 2. tight-junction marker (*Anti-ZO-1-Alexa Fluor 555* (MA3-39100-A555, Invitrogen)); 3. proliferating cell marker (*Anti-ki67-Alexa Fluor 488* (ab206633, Abcam)); 4. markers to identify differentiated/specialized cells (Goblet cells, colonocytes, enteroendocrine cells) (*Anti-Mucin2-Alexa Fluor 555* (bs-1993R-A555, Biocompare), *anti-Cytokeratin 20-Alexa Fluor 488* (ab275988, Abcam), *anti-Chromogranin A-Alexa Fluor 488* (ab199192, Abcam), respectively). Cell nuclei were labeled with the fluorescent dye *Hoechst 33342* (R37605, Invitrogen). Both brightfield and immunofluorescence microscopy of the samples were performed with 5 ×, 10 × and 40 × objectives using an inverted fluorescence microscope ZEISS Axio Observer with ZEISS ZEN 3.1 (blue edition) software.

### DNA isolation from biopsies, crypts, and organoids samples

DNA from fresh frozen biopsy samples, cryopreserved crypts and organoid specimens was extracted using *AllPrep DNA/RNA Mini Kit* (80204, Qiagen). Briefly, frozen biopsy samples were lysed on *MagNA Lyser* (Roche Diagnostics) (6000 rpm, twice for 15 s with a 15 s break) using *Lysing Matrix D* tubes (116913050-CF, MP Biomedicals) and 350 µl buffer RLT Plus. Cryopreserved pellets of crypts and organoids in *CryoStor® CS10* medium* (*07930, StemCell Technologies) were gently thawed at + 4 °C and centrifuged for 5 min at 400 × *g* at + 4 °C. Supernatant was removed and pellets was lysed in 350 µl buffer RLT Plus. The following steps of DNA extraction from the lysates of biopsies, crypts and organoids were performed in line with the manufacturer’s instructions.

### Bisulfite conversion and PCR Amplification

In total 200 ng of the isolated genomic DNA was bisulfite converted using *MethylCode™ Bisulfite Conversion Kit* (MECOV50, Applied Biosystems) and applied for 146 bp size LINE-1 region amplification via PCR. Samples containing no template were used for PCR contamination control. Custom-made primers set (F: 5′-TTTTGAGTTAGGTGTGGGATATA-3′, R: 5′-biotin-AAAATCAAAAAATTCCCTTTC-3′) (final concentration of each 0.2 µM) and *PyroMark® PCR Kit* (978703, Qiagen) was used for PCR amplification. Thermal-cycling conditions: 95 °C for 15 min; 45 cycles of 94 °C for 30 s, 56 °C for 30 s, 72 °C for 30 s; 72 °C for 10 min cycling mode. The specificity of PCR amplicon was verified using 2% agarose gel.

### LINE-1 methylation analyses

Methylation level of three CpG islands in amplified LINE-1 region was analyzed using PyroMark Q24 (Qiagen) pyrosequencing system. Briefly, 20 µl of PCR product was immobilized to *Streptavidin Sepharose HP beads* (17-5113-01, Cytiva), processed with the PyroMark Q24 Vacuum Workstation and annealed to the sequencing primer 5′-AGTTAGGTGTGGGATATAGT-3′. Sequence analysis was performed by applying *PyroMark Gold Q24 reagents* (970802, Qiagen). All samples were analyzed in duplicates. Positive control CpG *Methylated Human Genomic DNA* (SD1131, Thermo Scientific) and PCR negative control were used in each sequencing run. Methylation level of 60% and higher value was considered as high LINE-1 methylation based on the previous publications^25,29,30^.

### Statistical analysis

The pyrograms of LINE-1 region were analyzed using the *PyroMark Q24 software* (v. 2.0.8, Qiagen). Statistical analysis of methylation data results and data visualization were performed using *R studio* (R version 4.0.3) software and its packages (ggplot2, ggsignif, ggpubr, scales, base, stats, tidyverse). Differences between groups were considered statistically significant when the calculated p-value was equal to or lower than the critical level (*p* ≤ 0.05). The distribution of data in groups according to the normal (Gaussian) distribution was assessed by the Shapiro–Wilk normality test. Since the data in the study groups were not normally distributed, the Wilcoxon signed-rank test was used for statistical analysis.

### Supplementary Information

Supplementary Figure 1.

## Data Availability

The data presented in the current study are available on reasonable request from the corresponding author.
