# Supplementary material for: Prolonged culturing of colonic epithelial organoids derived from healthy individuals and ulcerative colitis patients results in the decrease of LINE-1 methylation level
Source: Sci Rep. 2024 Feb 23;14:4456. doi: 10.1038/s41598-024-55076-8 (PMC10891043; doi:10.1038/s41598-024-55076-8)
Supplement: Supplementary file 1 — Supplementary Figure 1. [file 41598_2024_55076_MOESM1_ESM.pdf]

Prolonged culturing of colonic epithelial organoids derived from healthy individuals and ulcerative colitis patients results in the decrease of LINE-1 methylation level

Inciuraite Ruta[1†], Steponaitiene Ruta[1†], Raudze Odeta[1], Kulokiene Ugne[1], Kiudelis Vytautas[2], Lukosevicius Rokas[1], Ugenskiene Rasa[3], Adamonis Kestutis[2], Kiudelis Gediminas[1,2], Jonaitis Laimas Virginijus[1,2], Kupcinskas Juozas[1,2], Skieceviciene Jurgita[1*]

[1] Institute for Digestive Research, Academy of Medicine, Lithuanian University of Health Sciences, A. Mickeviciaus st. 9, 44307, Kaunas, Lithuania

[2] Department of Gastroenterology, Academy of Medicine, Lithuanian University of Health Sciences, A. Mickeviciaus st. 9, 44307, Kaunas, Lithuania

[3] Department of Genetics and Molecular Medicine, Academy of Medicine, Lithuanian University of Health Sciences, A. Mickeviciaus st. 9, 44307, Kaunas, Lithuania

[†]These authors contributed equally to this work

*Corresponding author: Jurgita Skieceviciene, PhD, Institute for Digestive Research, Academy of Medicine, Lithuanian University of Health Sciences, Mickeviciaus st. 9, 44307, Kaunas, Lithuania. Email: jurgita.skieceviciene@lsmuni.lt, phone: (370 37) 327236.

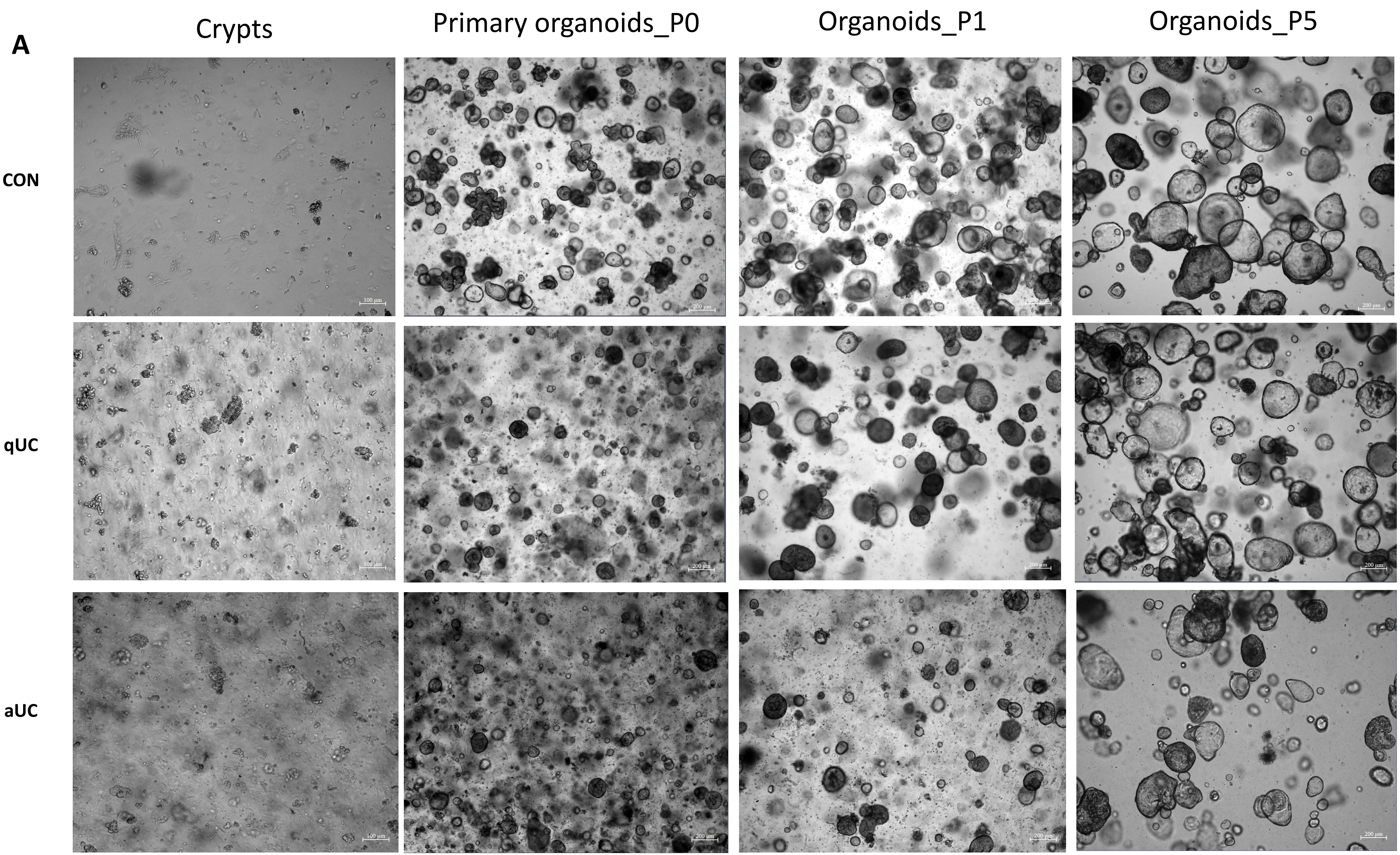

A
Crypts
Primary organoids_P0
Organoids_P1
Organoids_P5
CON
qUC
aUC

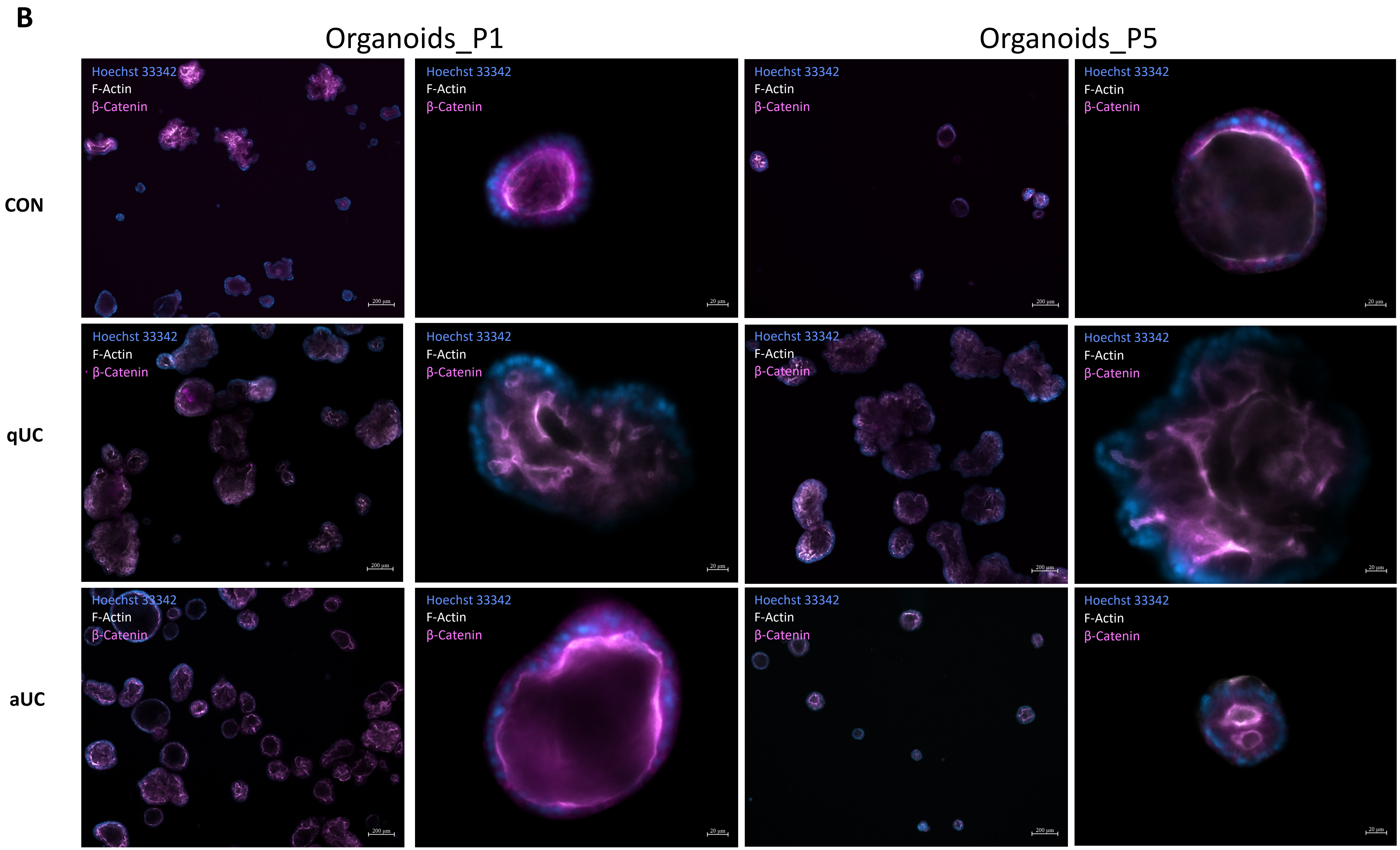
B
Organoids_P1
Organoids_P5
CON
qUC
aUC
Hoechst 33342
F-Actin
β-Catenin

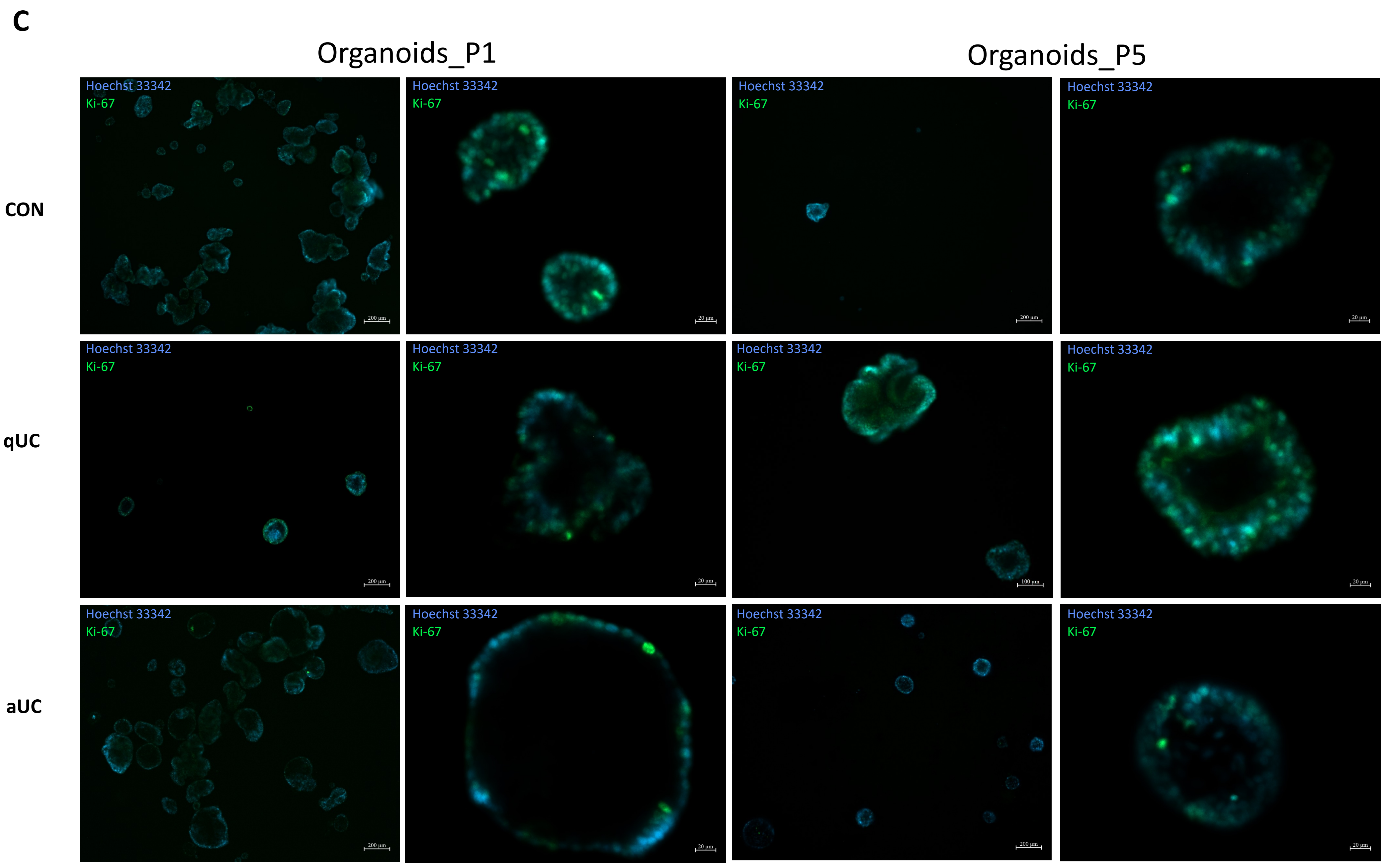
C
Organoids_P1
Organoids_P5
CON
qUC
aUC
Hoechst 33342
Ki-67
200 μm
20 μm
100 μm

D

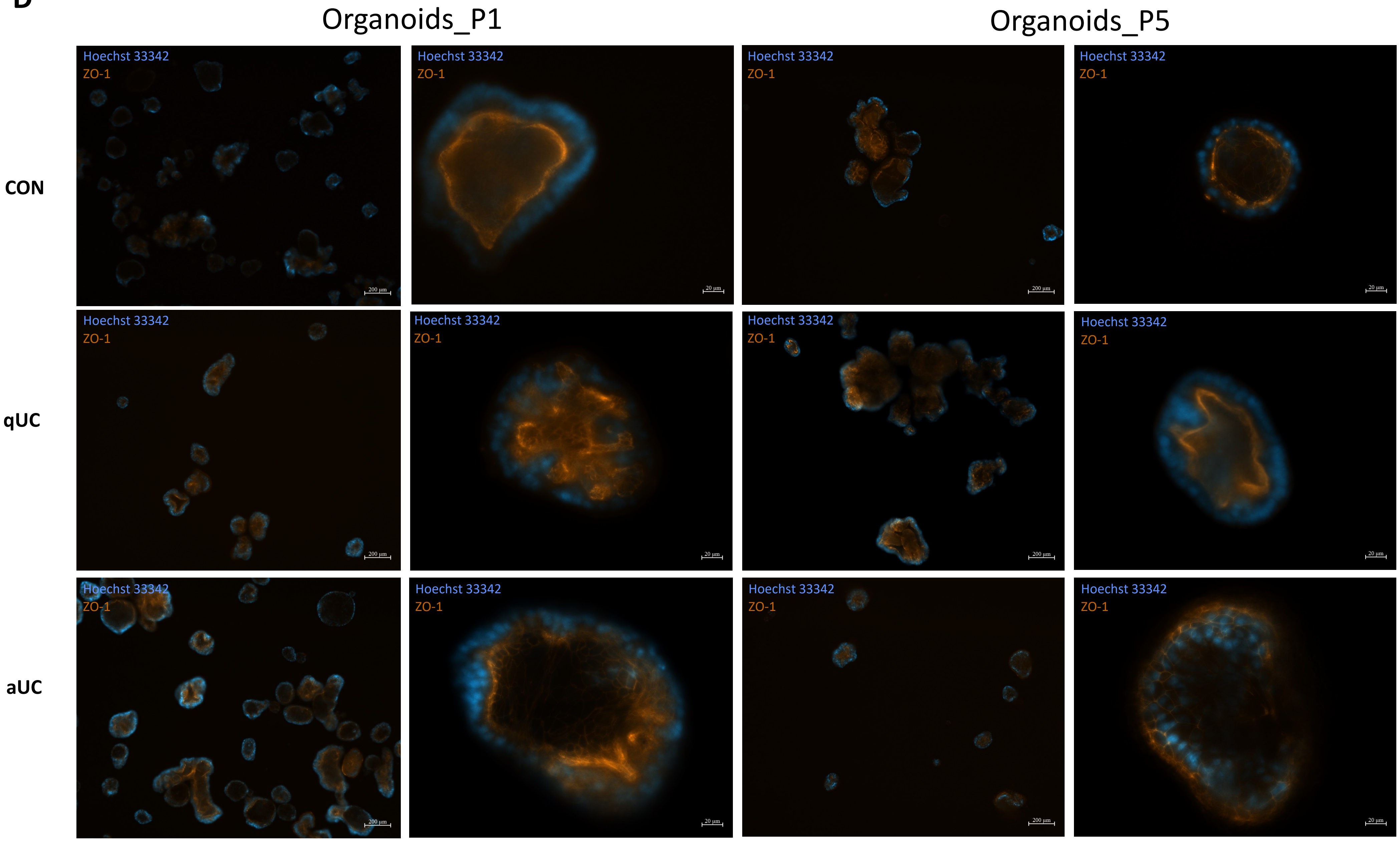

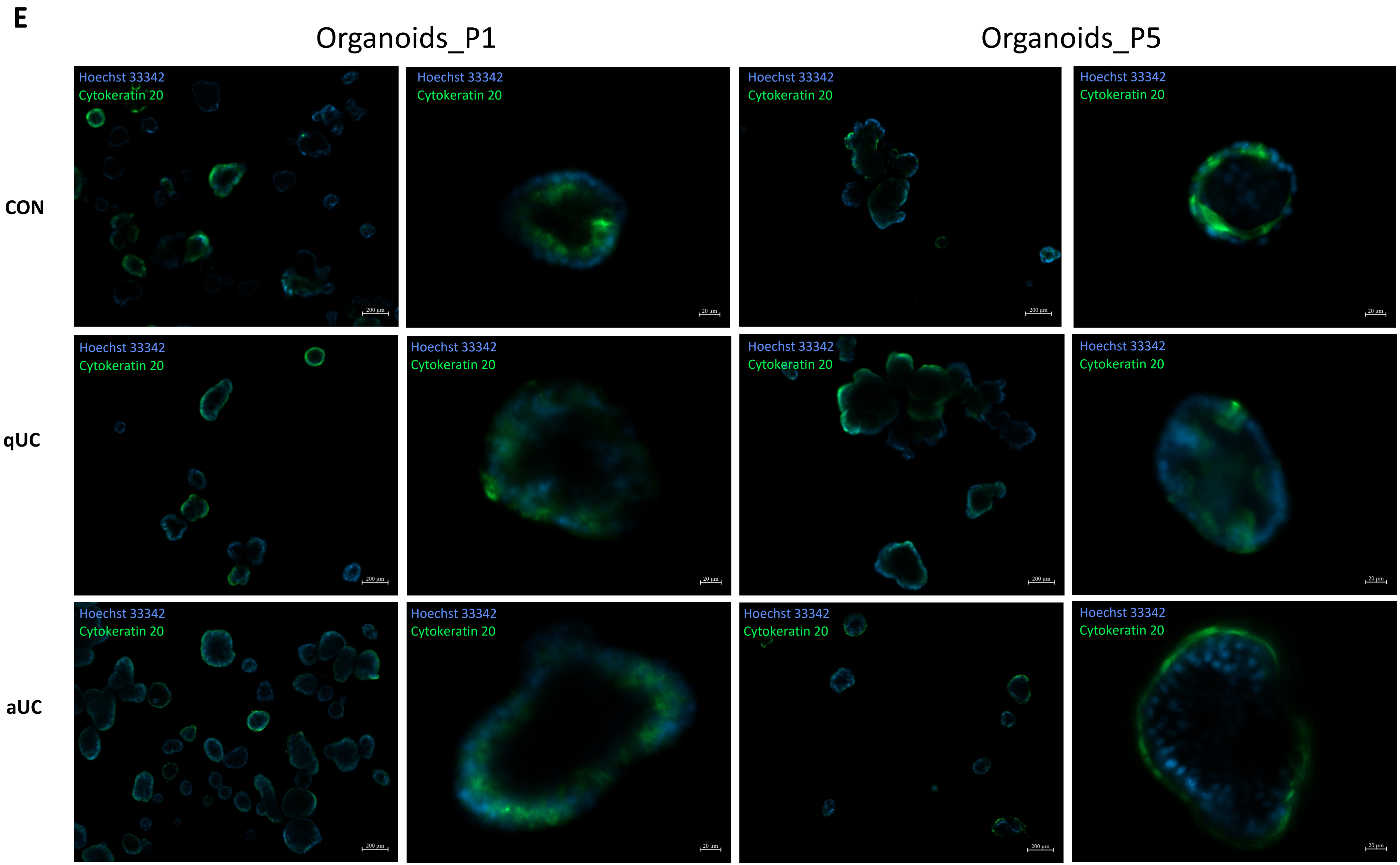
E
Organoids_P1
Organoids_P5
CON
qUC
aUC
Hoechst 33342
Cytokeratin 20

F

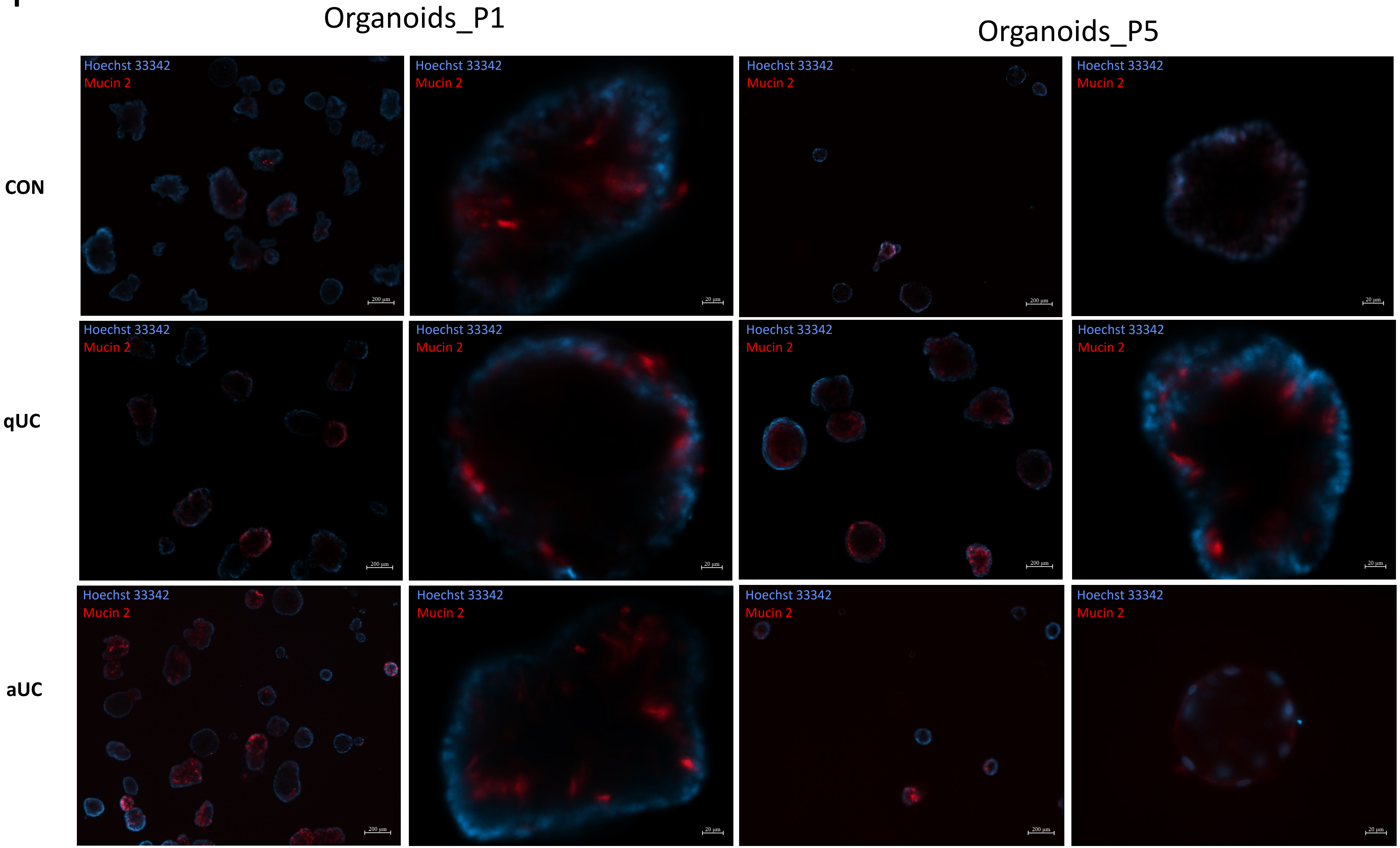

Organoids_P1
Organoids_P5
CON
qUC
aUC
Hoechst 33342
Mucin 2
200 μm
20 μm

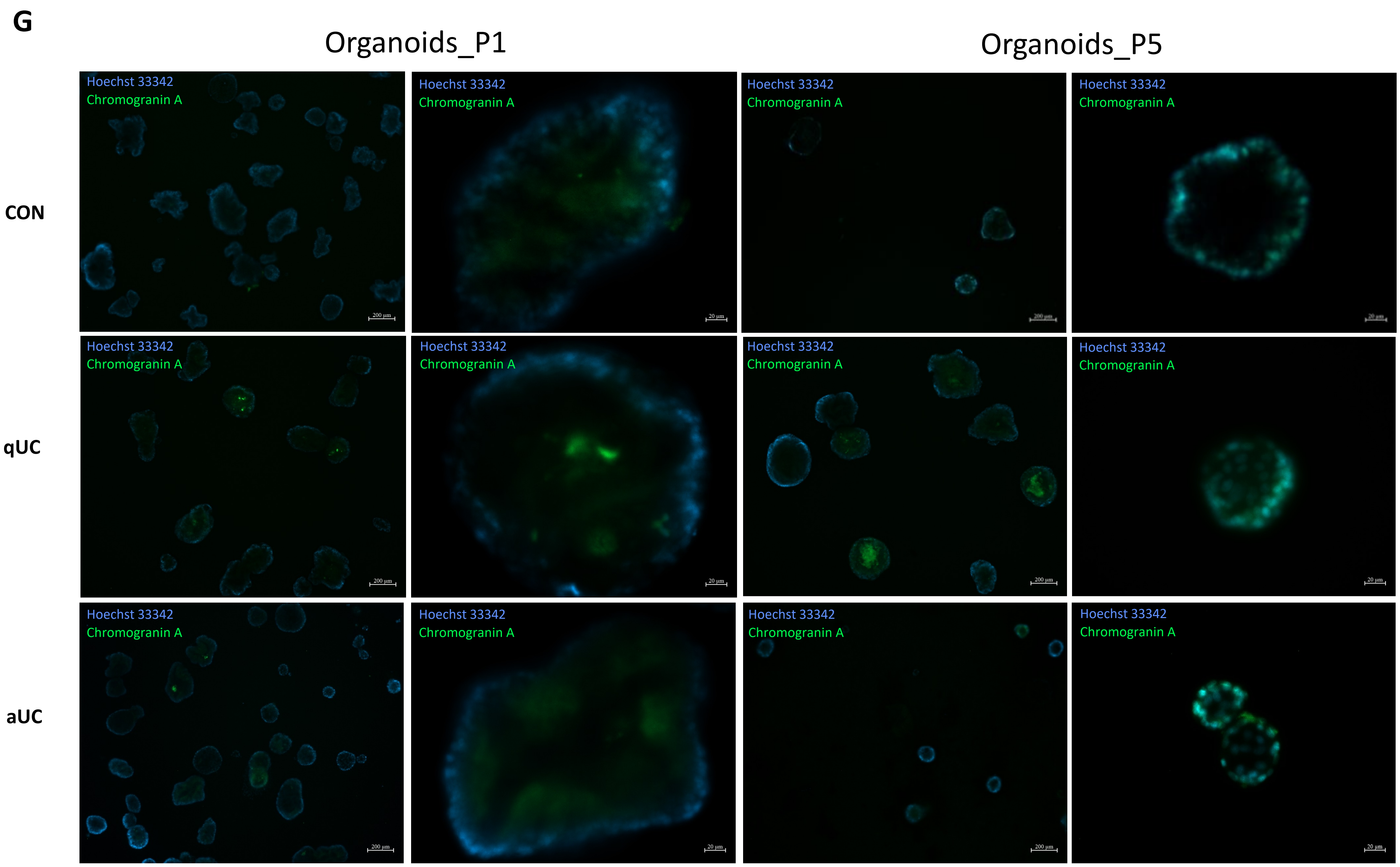
G
Organoids_P1
Organoids_P5
CON
qUC
aUC
Hoechst 33342
Chromogranin A

Supplementary Fig. 1. The dynamics of formation and structural rearrangements of UC patient- and control individual-derived human colon organoids during long-term culturing. All images were acquired with ZEISS Axio Observer 7 inverted fluorescence microscope using 5x, 10x and 40x objectives, and analyzed by ZEISS ZEN 3.1 (blue edition) software. **(A)** Representation of structural transition from freshly isolated UC patient and control individual colon crypts on left panel to early-passage (P0 and P1) cystic organoids in middle panels to late-passage (P5) cystic organoids on right panel. **(B-G)** Immunofluorescence microscopy pictures of early-passage (P1) and late-passage (P5) colon organoids showing consistent organoid structure and cellular composition. Hoechst 33342 (blue) was used in all cases as a counterstain for cell nuclei. **(B)** Proper epithelial cell monolayer polarity is defined by β-catenin (pink) labeling stained basolateral side and F-actin (Phalloidin) (white) labeling stained apical side. **(C)** Areas of proliferation are identified by Ki67 (green) expressing proliferating cells. **(D)** Epithelial barrier integrity is defined by detection of tight junction protein ZO-1 (orange). **(E)** Absorptive colonocytes are defined by positive Cytokeratin 20 (green) staining. **(F)** Mucin-producing Goblet cells are identified by positive Mucin 2 (red) staining. **(G)** Hormone-producing enteroendocrine cells are defined by positive Chromogranin A (green) staining. CON – control, qUC – quiescent ulcerative colitis, aUC – active ulcerative colitis.
